# Health service use of Australian unemployment and disability benefit recipients: a national, cross-sectional study

**DOI:** 10.1186/s12913-021-06255-0

**Published:** 2021-03-19

**Authors:** Alex Collie, Luke Sheehan, Ashley McAllister

**Affiliations:** 1 School of Public Health and Preventive Medicine, Monash University, 553 St Kilda Road, Melbourne, VIC 3004 Australia; 2 School of Population and Global Health, University of Melbourne, 207 Bouverie Street, Carlton, VIC 3053 Australia

**Keywords:** Health service use, Income support, Disability, Unemployment, Hospitalisation, Health professional consultations

## Abstract

**Background:**

Healthcare is funded and delivered separately from income support programs such as unemployment and disability benefits. Greater understanding of the health service use (HSU) of benefit recipients would support more effective design and delivery of health and income support programs. This study aimed to characterise the HSU of disability and unemployment benefit recipients relative to people earning wages, while controlling for personal, household and health-related factors associated with HSU in benefit recipients.

**Methods:**

A cross-sectional national survey of 9110 working age Australian adults in three groups: (1) 566 receiving the disability support pension (DSP); (2) 410 receiving unemployment benefits; and (3) 8134 earning wages. Outcomes included prevalence and frequency of health professional consultations, hospital attendance and admission in the past 12 months, as well as medication and supplement use in the past 2 weeks. Analyses compared DSP and unemployment benefit recipients to wage earners using prevalence ratios and incident rate ratios, adjusted for predisposing, enabling and need factors that may affect HSU.

**Results:**

In adjusted regression models, both DSP and unemployment benefit recipients were significantly more likely than wage earners to have consulted psychologists and social workers. DSP recipients also reported a significantly higher prevalence of consultations with General Practitioners (GPs), specialist physicians and podiatrists.. Both groups reported significantly more frequent consultations with GPs and DSP recipients with specialists. No differences were observed between groups for hospital attendance or admission, or supplement use in fully adjusted models, though the DSP group reported more prevalent medication use than wage earners. Inclusion of confounders including self-assessed health, disability severity, health insurance status, and financial resources attenuated the relationship between benefit receipt and HSU, however significant associations were still observed.

**Conclusions:**

People receiving unemployment and disability insurance benefits use significantly more health services than wage earners. A range of personal and clinical characteristics explained much, but not all, of the association between greater HSU and benefit receipt. Greater coordination between health and income support systems may improve health, reduce HSU and improve work ability in unemployed and working age people.

**Supplementary Information:**

The online version contains supplementary material available at 10.1186/s12913-021-06255-0.

## Background

In many nations, healthcare is delivered separately from government income support programs such as unemployment and disability benefits. This separation of function occurs despite substantial evidence that the two systems are inherently interconnected via the people they service. For example, unemployment and job loss is linked with poor health including higher mortality and morbidity [1, 2]. This study examines the health service use (HSU) of people receiving income support benefits, specifically the Australian means-tested welfare programs for people who are unemployed or living with disability.

### Relationship between health status and income support

Evidence shows that health status and receipt of government income support payments are inextricably linked. For example, an Australian study reported the prevalence of mental health conditions was significantly higher among those receiving disability pension compared with non-recipients [3], while another showed that the transition period from employment to disability pension receipt was linked with a decline in mental health [4]. A recent panel study identified that people with disability had higher levels of depression and anxiety symptoms during periods in which they were receiving disability benefits than when they were not, after accounting for time-varying changes in disability severity, suggesting a causal effect of benefit receipt on health [5]. These links are also observed in income support systems with differing eligibility criteria and benefits. For example, studies from the UK and northern Europe showed that restricting access to disability benefits contributed to growth in the number of people with significant health concerns enrolled in unemployment benefit programs [6], and increased the burden of ill health in this population [6–8]. In Sweden, people with frequent attendance in primary healthcare settings are more likely to receive disability benefits in future than non-attenders or those with infrequent attendance [9].

### The Australian context

In Australia, a growing portion of working age people receive government income support payments, in particular the means-tested disability benefit known as the Disability Support Pension (DSP) and the main means-tested unemployment benefit known as the Newstart Allowance (NSA). The DSP provides financial support to people aged 18 to 65 years with permanent physical, intellectual or psychiatric impairments that limit their ability to engage in employment. The NSA provided financial support to people who are unemployed, aged 22 years or older and whose income and assets were below set thresholds. From March 2020, the NSA was rolled into a more broadly defined benefit titled the Job Seeker Payment. Approximately 750,000 Australians, or 4.5% of the working age population, receive the DSP [10], while a further 1.3 million receive the NSA, of whom over 300,000 have been assessed as having medical conditions limiting their work capacity [11]. From herein we refer to these programs individually by their names or the associated abbreviation (DSP or NSA) or collectively as working-age benefits.

The Australian publicly funded healthcare system, known as Medicare, is largely disconnected from these benefit programs. While income support is funded through general taxation revenue, public healthcare is funded by a specific levy on income tax known as the ‘Medicare Levy’. Income support payments are administered by the federal government agency known as Centrelink, whereas healthcare services are delivered by state and territory governments, local governments and through private practices (e.g., privately operated general practice clinics). Eligibility for working-age benefits does not specifically confer eligibility for healthcare services, although people receiving the DSP and NSA do receive discounted health services and medicines. During the DSP application process individuals are required to collect and submit relevant medical information, and undergo medical examination by government appointed healthcare practitioners. This information is used solely for the purposes of determining eligibility, and not at all to review treatment or likely future service needs.

Despite the clear link between benefit receipt and health status, relatively little is known about the extent and patterns of HSU in working age benefit recipients. Poor health is a barrier to engagement in employment [12]. Effective health service delivery may support reductions in disability and improvements in work ability. An enhanced understanding of HSU among benefit recipients would therefore support the design and delivery of both health and social care.

### Study aims

This study aims to extend knowledge of the links between healthcare and government income support systems by characterising the HSU of working age benefit recipients. Specifically, the study aims to compare the health service use of DSP and NSA recipients to that of people earning wages or business income, while accounting for a range of personal, household and health-related factors that have been associated with HSU.

## Methods

### Study design and data source

This cross-sectional study utilises data from the Australian National Health Survey (NHS). The NHS is an Australia-wide health survey conducted every three years by the Australian Bureau of Statistics. This study utilises data from the NHS collected between July 2014 and July 2015 [13]. Data was collected on 19,259 individuals from 14,723 private dwellings by computer assisted personal interview. In some instances, adult respondents were unable to answer for themselves due to significant long-term illness or disability. In these cases, a person responsible for them was interviewed on their behalf and where possible, the respondent was present during the interview. The NHS collects data about demographic, socio-economic and health characteristics including; physical measurements, long-term health conditions, risk factors, and health-related actions taken including use of health services. Data collection was spread randomly over a 12-month period to account for seasonal health effects. The household response rate was 82%.

### Income support benefits

#### Disability support pension

The DSP is an income support benefit provided to Australian residents of working age whose disability or medical condition prevents them from working more than 15 h a week [14] . Applicants must have their primary medical condition assessed by a government-contracted doctor as lasting for at least 2 years, and as ‘fully diagnosed, treated and stabilised’. In addition, applicants must pass income and asset tests, and some applicants may be required to complete an 18-month work placement program that assesses their capacity for work. Once approved the DSP may be received until retirement age.

#### Newstart allowance and sickness allowance

The Newstart Allowance (NSA) and Sickness Allowance (SA) are mutually exclusive benefits (i.e., individuals cannot receive both at once) but are not able to be differentiated in the NHS data. Thus, both payments are described here. The Newstart Allowance (NSA) is a payment available for those aged 22 to retirement age that is designed to provide income support for unemployed individuals who are activity seeking employment. To receive the NSA individuals must pass income and asset tests, and then meet ongoing ‘mutual obligations’, such as provide evidence of ongoing job seeking efforts [15]. The Sickness Allowance (SA) is a payment available to employed people with injury or illness who have exhausted their employer provided benefits (e.g., sick leave, annual leave). The payment rate is equivalent to that of the NSA but is capped at 12 months duration. Income and assets test also apply to this benefit.

#### Comparison of benefits

The DSP is an ongoing payment, paid until retirement age once an application is approved, while NSA and SA recipients must actively engage with Centrelink to continue to receive payments. The standard rate of DSP was A$391.10 per week in March 2015, while the standard NSA/SA payment was A$263.80 per week (the standard rates refer to a single recipient with no dependent children) [16]. Individuals who are in the process of applying for the DSP or have had their DSP claims rejected are likely to be receiving the NSA, as access is not disability dependent. The DSP fortnightly income cut-off for a single adult aged 21 years or older is AUD$2066.60 (as at January 2021) or $3163.20 for an adult couple living together. For a single homeowner DSP payments reduce when assets exceed $268,000, or $482,500 for a single non-homeowner. For a single person with no children NSA payments cease when fortnightly income exceeds $1257.50 while assets tests are equivalent to those for the DSP.

### Participants

As DSP and NSA payments are only available to persons of working age, the sample was first restricted to those aged 18 to 64 years. The NHS included questions on the income sources of respondents, including government benefits. This data was used to define three groups of respondents:

- *Group 1 – Disability Support Pension Recipients*

Respondents who reported their primary income source being the DSP.

- *Group 2 – Newstart Allowance Recipients*

Respondents who reported that they currently receive the NSA or the SA. During the survey collection period there were 91–95 times more Australians receiving the NSA benefit than there were receiving the SA benefit at each of the quarters with data was reported (September 2014, December 2014, March 2015, and June 2015) [17]. This group is therefore highly likely to be comprised of mainly NSA recipients.

- *Group 3 – Wage Earners*

Respondents indicating current income source as wages, business income or other cash income. Participants were excluded from this group if they received any federal government benefits or payments, with the exception of the Family Tax Benefit, which is widely available to Australian families with children.

### Outcomes

Three outcome categories were defined using the health service data recorded in the NHS. These were:

1. *Health Professional Consultations in past 12 Months*

This included (a) binary (yes/no) indicators of whether the respondent has consulted with a range of medical, allied and community health care practitioners in the past 12 months (list provided in Supplementary Material), and (b) the frequency of consultations with a General Practitioner (GP), dentist, or specialist medical practitioner in the past 12 months.

2. *Hospital attendance or admission in past 12 months*

This included (a) binary (yes/no) indicators of whether a participant had attended an emergency department or been admitted to hospital in the past 12 months, and (b) the frequency of emergency department attendance and hospital admissions in the past 12 months.

3. *Medication and Supplement use in past 2 Weeks*

This included (a) binary (yes/no) indicators of whether a participant had used any medications or any supplements in the past 2 weeks; and (b) the number of different types of medications and supplements reported as being used in the past 2 weeks.

Further details of data collection are provided in Supplementary Digital Material.

### Covariates

Covariate selection was guided by use of the Andersen and Newman Behavioural Model of Health Services Use [18]. Available variables were chosen that best captured the ‘Population Characteristics’ that lead to ‘Health Behaviour’, including predisposing, enabling and need factors.

#### Predisposing factors

Demographic factors included age and sex. Age was categorised into 10-year brackets (except for 18–24 years). Sex was recorded as either male or female. Education was selected as an indicator of social structure. Highest level of education was classified as ‘Less than Year 12’, ‘Year 12’, ‘Certificate or Diploma’, ‘Bachelor Degree’ and, ‘Postgraduate Degree’. No variables related to health beliefs were available in the NHS dataset and so this remained unmeasured.

#### Enabling factors

Personal/family factors were measured using insured status and financial reserves. Private health insurance status was analysed as a binary yes/no variable. Access to financial resources was assessed using the question ‘If all of a sudden you/this household had to get $2000 for something important, could the money be obtained within a week?’, responses were either yes or no. Community was measured using remoteness, mapped to the Accessibility and Remoteness Index of Australia, with the options of ‘Major cities’, ‘Inner regional’, and ‘Other’. ‘Other’ is an amalgam of ‘Outer Regional’, ‘Remote’ and ‘Very Remote’ which were combined in the provided data set due their relative infrequency.

#### Need factors

Perceived need was assessed using self-assessed health and disability status. Self-assessed health was measured with the question ‘In general would say that your health is excellent, very good, good, fair or poor?’ and the Likert response scale was used for analysis. Disability status was coded by the ABS based on responses regarding disabilities or long-term health conditions and how they impacted the individual’s core activities (mobility, self-care and communication). Five levels of activity limitation were determined; profound, severe, moderate, mild, and school/employment restriction only. For analysis profound and severe were combined, and mild and moderate were combined. The total number of health conditions that each participant reported was used as a proxy for evaluated need. Although these conditions were self-reported by the participants, they were specifically asked whether the condition had been diagnosed and if the condition was current and long-term.

### Data analysis

#### Descriptive analysis

Counts and percentages were used to describe the characteristics of the sample, as all variables were categorical. Counts and percentages were also used to describe the prevalence of health outcomes within each income group. The frequency of each health service use outcome and number of medications and supplements taken was reported using mean and standard deviation.

#### Regression analysis

Robust Poisson regression models with each HSU and medication/supplement use as an outcome and income group as the exposure variable were performed to estimate prevalence ratios (PRs) and 95% confidence intervals (CIs). ‘Base’ models that were adjusted for age and sex were calculated to characterise differences between income groups. ‘Fully Adjusted’ robust Poisson models were then run with all covariates included.

Negative binomial regression models with the frequency of each HSU and medication/supplement use as an outcome and income group as the exposure variable were utilised to estimate incidence rate ratios (IRRs) with 95% confidence intervals. ‘Base’ models that were adjusted for age and sex were calculated to characterise differences between income groups. ‘Fully Adjusted’ negative binomial models were then run with the remaining covariates included.

## Results

### Overview of participants

A total of 11,296 working age adults completed the NHS. Of these 566 met criteria for inclusion in the DSP recipient group, 410 to the NSA group and 8134 to the wage earners group. The remaining 2186 individuals of working age participated in the NHS but did not fit the inclusion criteria of any group, and were excluded from analyses.

The DSP group was older than the other groups (Table 1). Females comprised 49, 54, and 57% of the wage earners, DSP and NSA groups, respectively. Differences in education and socioeconomic status were also observed.

**Table 1:** Characteristics of study groups

	Wage Earners (Column %)	DSP Group (Column %)	NSA Group (Column %)
Total Number	8134 (100%)	566 (100%)	410 (100%)
Age in Years			
18 to 24	707 (8.7%)	17 (3.0%)	30 (7.3%)
25 to 34	1873 (23.0%)	27 (4.8%)	65 (15.9%)
35 to 44	2048 (25.2%)	85 (15.0%)	103 (25.1%)
45 to 54	1893 (23.3%)	159 (28.1%)	103 (25.1%)
55 to 64	1613 (19.8%)	278 (49.1%)	109 (26.6%)
Sex			
Male	4154 (51.1%)	258 (45.6%)	177 (43.2%)
Female	3980 (48.9%)	308 (54.4%)	233 (56.8%)
Highest Level of Education			
Less than Year 12	1192 (14.7%)	298 (52.7%)	167 (40.7%)
Year 12	1082 (13.3%)	72 (12.7%)	43 (10.5%)
Certificate or Diploma	2854 (35.1%)	153 (27.0%)	151 (36.8%)
Bachelor Degree	1922 (23.6%)	36 (6.4%)	34 (8.3%)
Postgraduate Degree	1084 (13.3%)	7 (1.2%)	15 (3.7%)
Private Health Insurance Status			
With private health insurance	5441 (66.9%)	92 (16.3%)	79 (19.3%)
Without private health insurance	2693 (33.1%)	474 (83.7%)	331 (80.7%)
Whether Household could Raise $2000 in an Emergency			
No	632 (7.8%)	304 (53.7%)	214 (52.2%)
Yes	7502 (92.2%)	262 (46.3%)	196 (47.8%)
Remoteness			
Major Cities of Australia	5597 (68.8%)	328 (58.0%)	233 (56.8%)
Inner regional Australia	1317 (16.2%)	132 (23.3%)	102 (24.9%)
Other	1220 (15.0%)	106 (18.7%)	75 (18.3%)
Self-Assessed Health			
Excellent	1849 (22.7%)	16 (2.8%)	43 (10.5%)
Very good	3314 (40.7%)	44 (7.8%)	109 (26.6%)
Good	2284 (28.1%)	156 (27.6%)	122 (29.8%)
Fair	571 (7.0%)	188 (33.2%)	95 (23.2%)
Poor	116 (1.4%)	162 (28.6%)	41 (10.0%)
Disability Status			
No disability or long-term health condition	5944 (73.1%)	65 (11.5%)	190 (46.3%)
No limitation or specific restriction	1210 (14.9%)	16 (2.8%)	51 (12.4%)
Schooling/employment restriction only	283 (3.5%)	72 (12.7%)	54 (13.2%)
Mild/moderate core activity limitation	591 (7.3%)	248 (43.8%)	97 (23.7%)
Severe/profound core activity limitation	106 (1.3%)	165 (29.2%)	18 (4.4%)
Number of ICD-10 Conditions			
0–4	5850 (71.9%)	116 (20.5%)	203 (49.5%)
5–9	1964 (24.1%)	217 (38.3%)	135 (32.9%)
10–14	275 (3.4%)	149 (26.3%)	56 (13.7%)
15+	45 (0.6%)	84 (14.8%)	16 (3.9%)

Note: All data is presented as Number (column percentage); *DSP* Disability Support Pension, *NSA* Newstart Allowance

Less than one in five DSP and NSA recipients had private health insurance, compared to two thirds of wage earners. Ninety-two percent of wage earners reported that their household could raise $2000 within a week, compared to 46 and 48% for DSP and NSA recipients, respectively. Wage earners had a higher proportion of respondents in major cities than DSP and NSA recipients. More than 90% of wage earners rated their self-assessed health as excellent, very good, or good, compared with 39.2% of DSP recipients; and 66.9% of NSA recipients.

Twenty-nine percent of DSP recipients reported severe core activity limitation and 44% mild/moderate limitation. Just under three quarters of wage earners reported no disability or long-term health condition. In the NSA group, 47% reported no disability or long-term health condition, 24% reported a mild/moderate core activity limitation and 4% severe limitation. Seventy-nine percent of DSP recipients reported at least five health conditions, compared to 50% of NSA recipients and 28% of wage earners. DSP recipients were also most likely to report over 15 conditions (15%), compared to 4 and 1% in NSA recipients and wage earners, respectively.

### Health professional consultations

More than 80% of respondents in all groups consulted a GP at least once in the previous 12 months. The next most commonly reported healthcare practitioners were specialists and dentists (Table 2), noting that GPs, specialists and dentists were the only health professionals where respondents were prompted by the interviewer to report consultations (see Methods).

**Table 2:** Prevalence of health professional consultations, hospital attendance/admission and medication/supplement use by study group

	Wage Earners Group	DSP Group	NSA Group
N Respondents	8134 (100%)	566 (100%)	410 (100%)
Health Professionals			
General Practitioner	6857 (84.3%)	541 (95.6%)	362 (88.3%)
Specialist	2656 (32.7%)	345 (61.0%)	159 (38.8%)
Dentist	4014 (49.3%)	203 (35.9%)	150 (36.6%)
Chemist (for advice only)	736 (9.0%)	137 (24.2%)	50 (12.2%)
Psychologist	408 (5.0%)	105 (18.6%)	52 (12.7%)
Other Health Professional	416 (5.1%)	78 (13.8%)	29 (7.1%)
Optician/Optometrist/Orthoptist	557 (6.8%)	77 (13.6%)	16 (3.9%)
Nurse	263 (3.2%)	73 (12.9%)	28 (6.8%)
Physiotherapist/Hydrotherapist	823 (10.1%)	68 (12.0%)	23 (5.6%)
Radiographer	371 (4.6%)	62 (11.0%)	26 (6.3%)
Dietitian/Nutritionist	176 (2.2%)	57 (10.1%)	12 (2.9%)
Podiatrist	235 (2.9%)	46 (8.1%)	9 (2.2%)
Social Worker/Welfare Officer	46 (0.6%)	43 (7.6%)	18 (4.4%)
Diabetes Educator	78 (1.0%)	39 (6.9%)	6 (1.5%)
Counsellor	143 (1.8%)	37 (6.5%)	20 (4.9%)
Occupational Therapist	72 (0.9%)	22 (3.9%)	5 (1.2%)
Chiropractor	504 (6.2%)	17 (3.0%)	13 (3.2%)
Audiologist/Audiometrist	49 (0.6%)	14 (2.5%)	< 5
Sonographer	109 (1.3%)	12 (2.1%)	5 (1.2%)
Acupuncturist	186 (2.3%)	10 (1.8%)	< 5
Naturopath	198 (2.4%)	9 (1.6%)	8 (2.0%)
Osteopath	144 (1.8%)	< 5	< 5
Hospital Admission / Attendance			
Hospital Admission	795 (9.8%)	141 (24.9%)	67 (16.3%)
Emergency Presentation	838 (10.3%)	129 (22.8%)	67 (16.3%)
Medications and Supplements			
Medications	3743 (46.0%)	491 (86.7%)	232 (56.6%)
Supplements	3485 (42.8%)	230 (40.6%)	147 (35.9%)

Note: Data represent the number (column percentage) participants in each group who consulted a health professional or health centre in the previous 12 months and the number (column percentage) who took a medication or supplement in the previous 2 weeks. *DSP* Disability Support Pension, *NSA* Newstart Allowance. Count data has been suppressed in cells with fewer than 5 cases (presented as < 5)

**Fig. 1:**
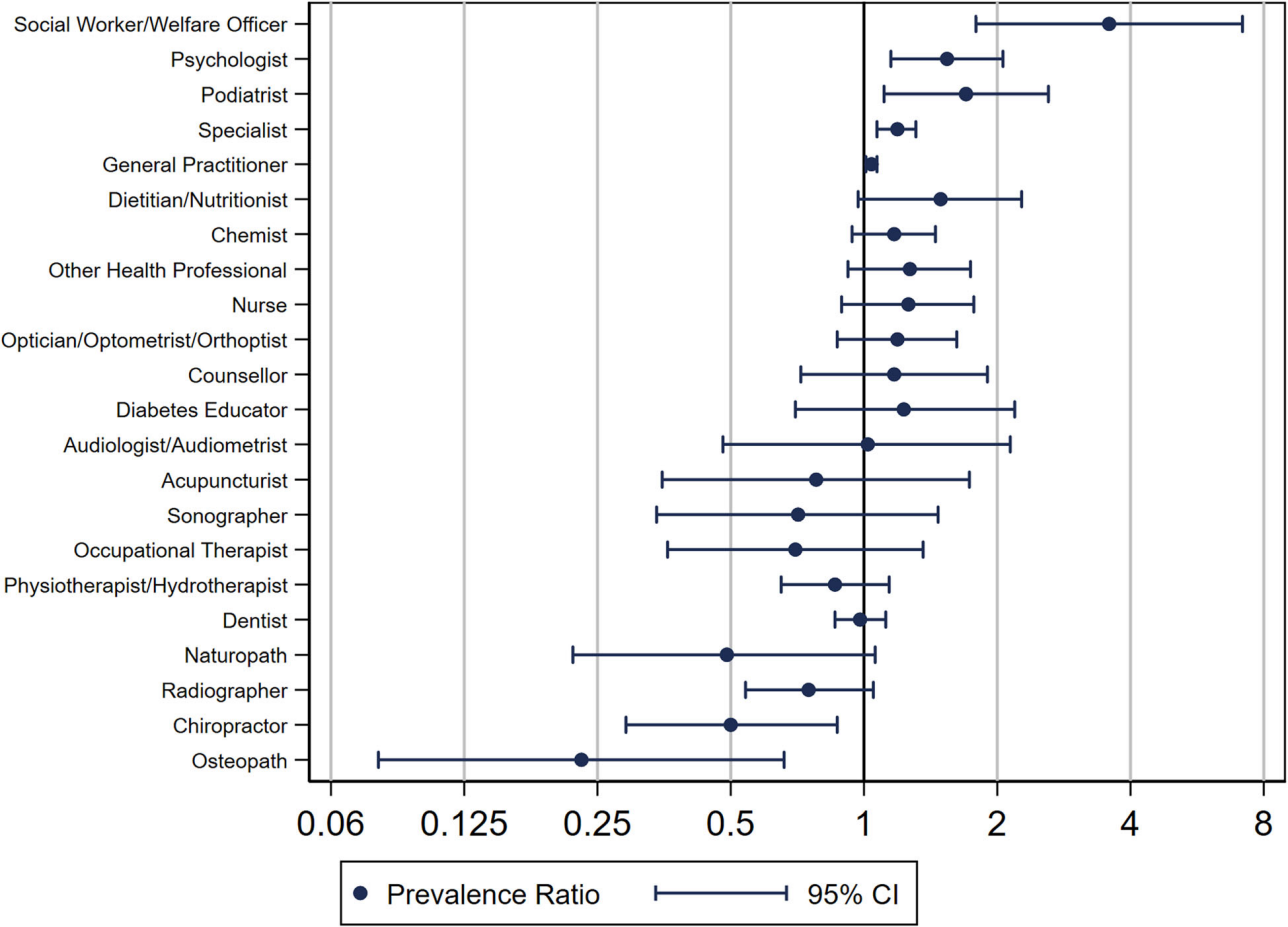
Adjusted prevalence ratios for having consulted health professionals in the past 12 months for disability benefit recipients compared to wage earners. CI: Confidence Interval

After adjustment for age and sex, DSP recipients were significantly more likely than wage earners to have consulted 15 of the 22 types of health professionals and significantly less likely to have consulted three (Supplementary Table 1). In fully adjusted models DSP recipients were significantly more likely than wage earners to have consulted five types of health professionals including social workers, psychologists, podiatrists, specialists and GPs, and significantly less likely to have consulted chiropractors and osteopaths (Fig. 1 and Supplementary Table 1).

**Fig. 2:**
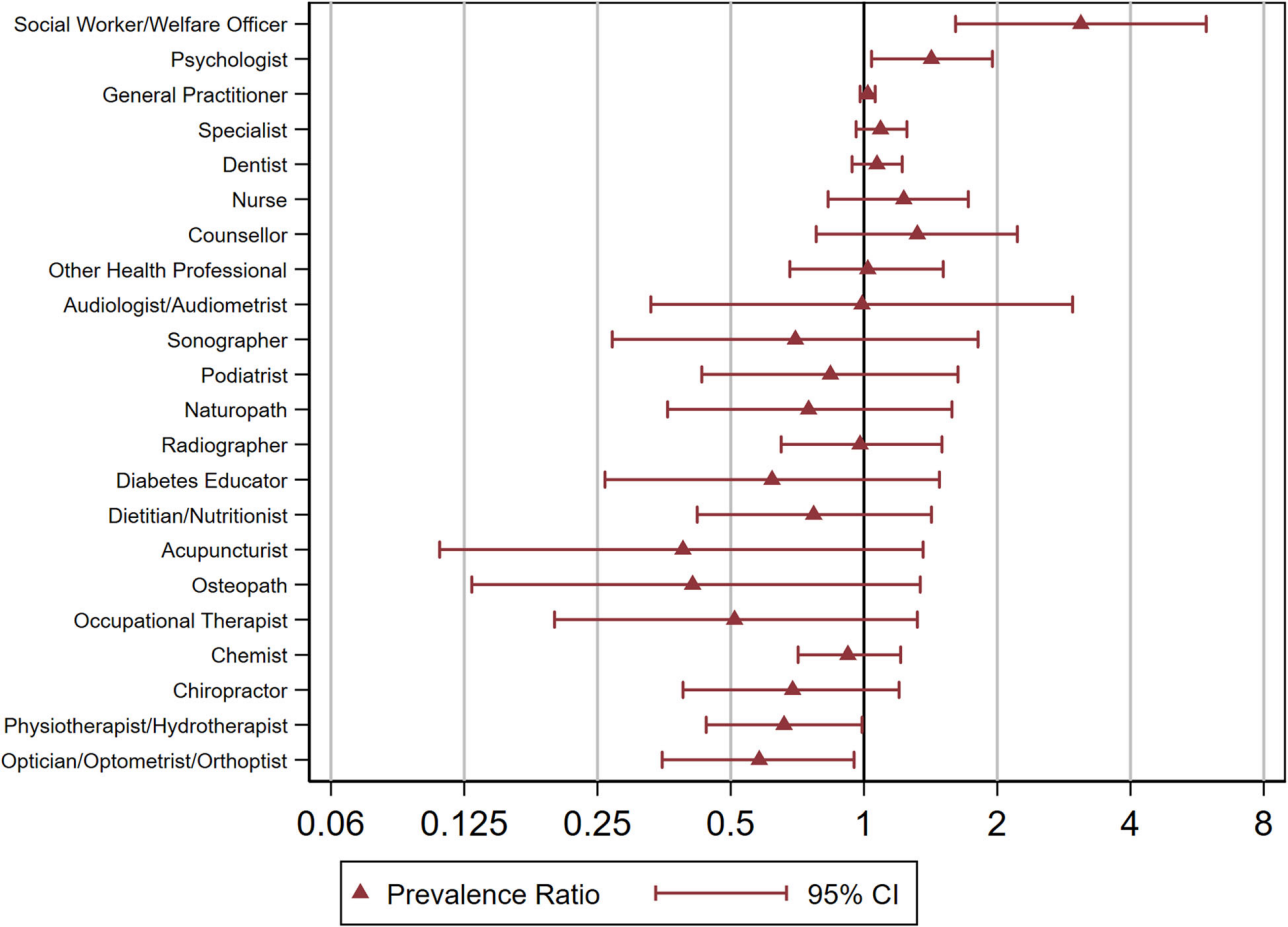
Adjusted prevalence ratios for having consulted health professionals in the past 12 months for unemployment benefit recipients compared to wage earners. CI: Confidence Interval

In the baseline models adjusted for age and sex, NSA recipients were significantly more likely than wage earners to have consulted five types of health professionals and significantly less likely to have consulted four types (Supplementary Table 2). In fully adjusted models NSA recipients were significantly more likely than wage earners to have consulted a social worker or psychologist and significantly less likely to have consulted an optician or a physiotherapist (Fig. 2 and Supplementary Table 2).

In both the DSP and NSA groups, the largest differences from wage earners were observed for consultations with a social worker or welfare officer, noting the low prevalence of consultations with these professionals in the wage earners group.

DSP recipients reported consulting a GP an average of 7.7 times in the past 12 months, compared with 3.1 times for wage earners and 5.0 times for NSA recipients (Table 3). DSP recipients visited a specialist a mean of 3.0 times in 12 months, compared to means of 1.0 and 1.3 times for wage earners and NSA recipients, respectively. Dental consultations averaged close to once a year in all three groups.

**Table 3:** Frequency of health professional consultations, hospital admission/attendance and medication/supplement use by study group

	Wage Earners Mean (SD)	DSP Group Mean (SD)	NSA Group Mean (SD)
Health Professionals			
General Practitioner	3.1 (3.0)	7.7 (4.2)	5.0 (4.1)
Specialist	1.0 (2.2)	3.0 (3.9)	1.3 (2.6)
Dentist	1.0 (1.6)	1.1 (2.3)	0.8 (1.7)
Hospital Admission / Attendance			
Hospital Admission	0.1 (0.5)	0.5 (1.3)	0.3 (0.7)
Emergency Presentation	0.1 (0.6)	0.6 (1.6)	0.3 (0.7)
Medications and Supplements			
Medications	0.9 (1.4)	3.9 (3.1)	1.6 (2.1)
Supplements	0.9 (1.3)	0.8 (1.3)	0.7 (1.3)

Note: Data represent the mean (standard deviation) number of visits to a health professional or health centre in the previous 12 months, and the mean (standard deviation) of use of different medications or supplements in the previous 2 weeks. *DSP* Disability Support Pension, *NSA* Newstart Allowance, *SD* Standard Deviation

**Fig. 3:**
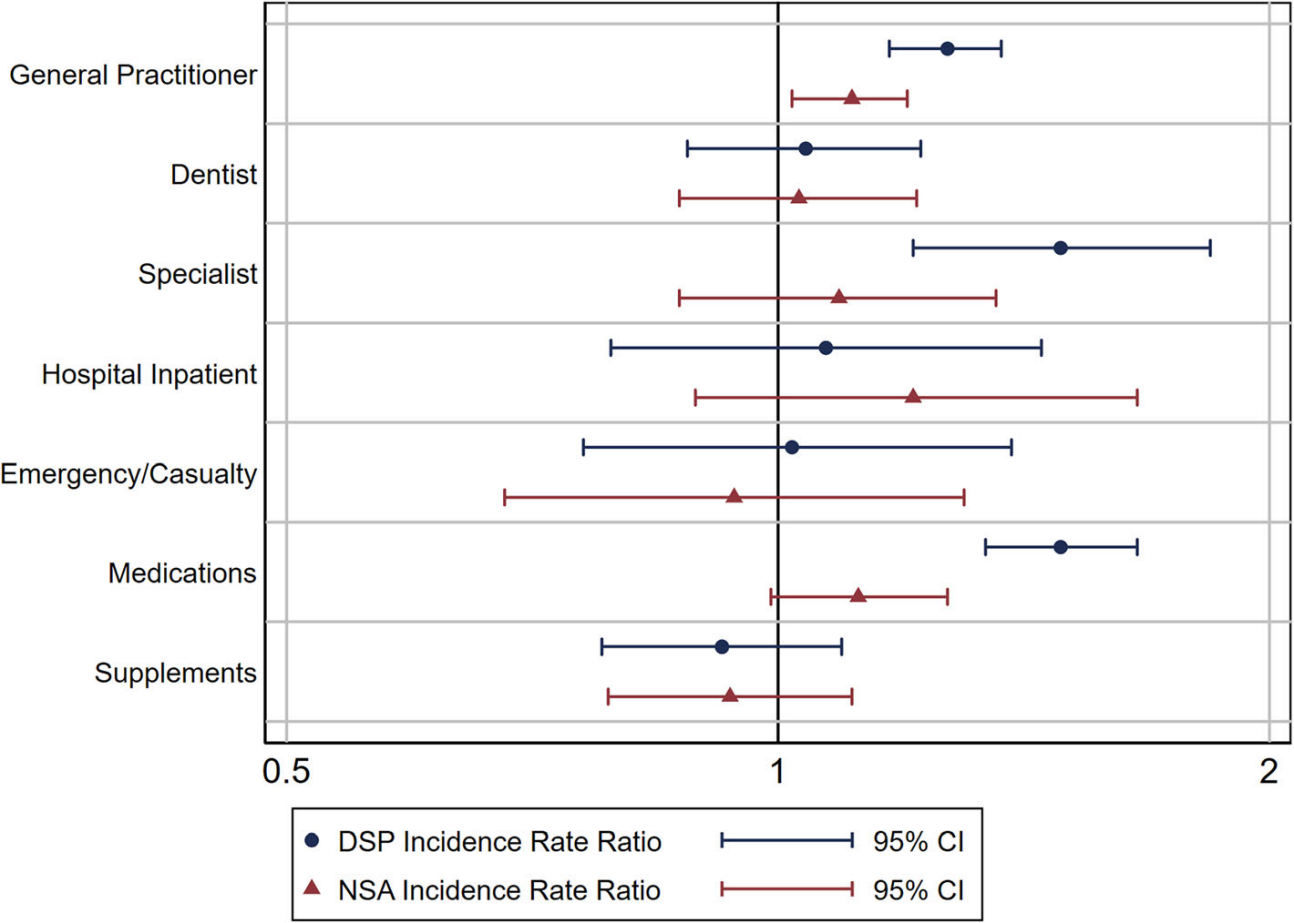
Adjusted incidence rate ratios for **a**) the frequency health professional consultations or hospital admission in the past 12 months and **b**) the number of different medication or supplements taken in the past two weeks, relative to wage earners. CI: Confidence Interval, DSP: Disability Support Pension, NSA: Newstart Allowance

Age and sex adjusted IRRs demonstrated that the differences between groups for GPs and specialists were significant, with both DSP and NSA groups reporting greater frequency than wage earners (Supplementary Table 3). After adjustment for all covariates (Fig. 3 and Supplementary Table 3) significant differences remained between DSP recipients in comparison to wage earners for frequency of GP consultations (IRR: 1.27, 95% CI: 1.17–1.37) and for consultations with a specialist (IRR: 1.49, 95% CI: 1.21–1.84). Frequency of GP consultations also remained significantly higher in NSA recipients compared to wage earners in fully adjusted models, with an IRR of 1.11 (95% CI: 1.02–1.20).

### Hospital attendance and admission

**Fig. 4:**
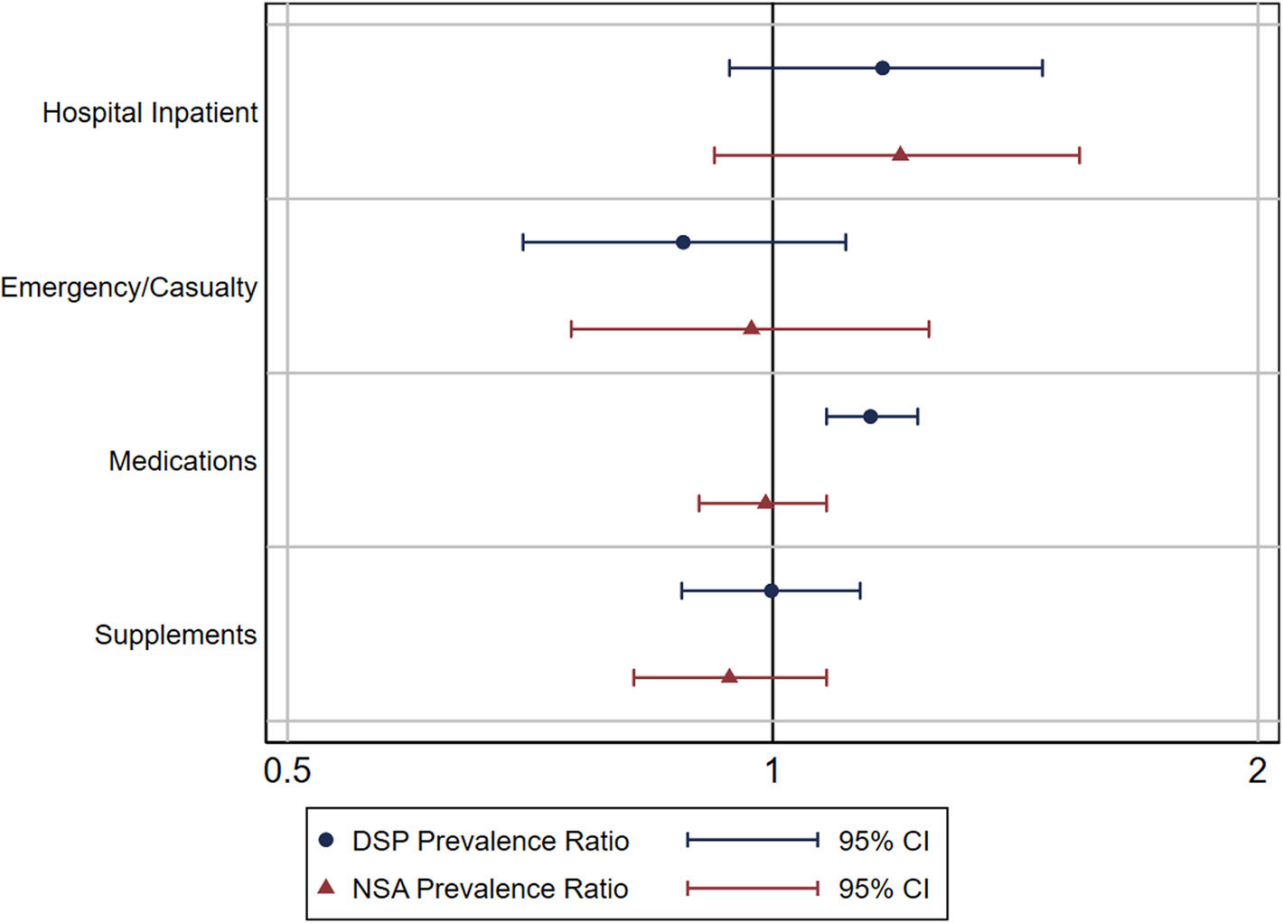
Adjusted prevalence ratios for **a**) having used a particular health service at least once in the past 12 months and **b**) having taken at least one medication or at least one supplement in the previous two weeks, relative to wage earners. CI: Confidence Interval, DSP: Disability Support Pension, NSA: Newstart Allowance

A greater proportion of DSP recipients reported attending an emergency department (23.2%) or being admitted to hospital as an inpatient (25.7%) at least once in the past 12 months than either the NSA and wage earner groups (Table 2). The age and sex adjusted PRs for DSP recipients compared to wage earners were 2.35 (95% CI: 1.98–2.79) for attending an emergency department and 2.46 (95% CI: 2.08–2.89) for hospital admission (Supplementary Table 4). Age and sex adjusted PRs for NSA recipients compared to wage earners were 1.61 (95% CI: 1.28–2.02) for attending an emergency department and 1.63 (95% CI: 1.30–2.06) for hospital admission (Supplementary Table 4). After all confounders were added to regression models, the differences in prevalence were non-significant for both DSP and NSA groups (Fig. 4 and Supplementary Table 4).

DSP recipients had a mean number of hospital attendance and admission of 0.5 in the past 12 months, NSA recipients of 0.3 and wage earners of 0.1 (Table 3). For emergency department presentations, the means were 0.6 for DSP recipients, 0.3 for NSA recipients and 0.1 for wage earners. Age and sex adjusted IRRs for DSP recipients compared to wage earners were 3.74 (95% CI: 2.97–4.71) for hospital admission as an inpatient and 4.51 (95% CI: 3.55–5.74) for attending an emergency department (Supplementary Table 3). Age and sex adjusted IRRs for NSA recipients compared to wage earners were 1.76 (95% CI: 1.30–2.39) for attending emergency and 1.96 (95% CI: 1.47–2.62) for inpatient admission.

In fully adjusted models the IRRs for hospital admission for DSP and NSA recipients were not significantly different to wage earners (Fig. 3 and Supplementary Table 3). There were also no significant differences in the IRRs for emergency department presentations between groups in fully adjusted models.

### Medication and supplement use

Eighty-six percent of DSP recipients reported having taken a medication in the previous two weeks in comparison to 57% of NSA recipients and 46% of wage earners (Table 2). In contrast, wage earners were the most likely to report supplement use in the past two weeks. After age and sex adjustment the PR for DSP recipients for medication use was 1.64 (95% CI: 1.57–1.71) and for NSA recipients 1.17 (95% CI: 1.07–1.27) in reference to wage earners (Supplementary Table 4). For supplement use the risk was significantly lower for both DSP (PR: 0.88, 95% CI: 0.79–0.97) and NSA recipients (PR: 0.80, 95% CI: 0.70–0.91) in reference to wage earners. After the addition of the remaining confounders prevalence of medicine use remained significantly greater in the DSP group with a PR of 1.15 (95% CI: 1.08–1.23). Prevalence of both medicine and supplement use in NSA recipients and supplement use in DSP recipients attenuated towards parity with wage earners in fully adjusted models (Fig. 4 and Supplementary Table 4).

DSP recipients reported using 3.9 different medicines on average in the past two weeks, compared to 1.6 for NSA recipients and 0.9 for wage earners. In contrast, wage earners reported taking the most supplements, with an average of 0.9, slightly more than DSP and NSA recipients with 0.8 and 0.7, respectively (Table 3). After age and sex adjustment the IRRs for medication use in reference to wage earners was 3.78 (95% CI: 3.07–3.72) for DSP recipients and 1.61 (95% CI: 1.42–1.82) for NSA recipients (Supplementary Table 3). The age and sex adjusted IRRs for supplement use was 0.81 (95% CI: 0.71–0.93) for DSP recipients and 0.77 (95% CI: 0.66–0.91) for NSA recipients. In fully adjusted models DSP recipients had a higher IRR of medication use (IRR: 1.49, 95% CI: 1.34–1.66), while NSA recipients had a slightly higher but non-significant IRR of 1.12 (95% CI: 0.99–1.27). Incidence of supplement use was slightly lower in the DSP and NSA recipient groups but were not significantly different from the wage earners group after adjustment (Fig. 3 and Supplementary Table 3).

### Effect of confounders

Inclusion of confounders assessing need and enabling aspects of the Andersen and Newman framework in fully adjusted models reduced the number and magnitude of significant associations observed between income support benefit group and health service use (Supplementary Tables 1 to 4). For DSP recipients, we observed the prevalence of HSU was statistically significantly associated with benefit status in 18 of 22 health professional types in models adjusted for age and sex. This reduced to 7 of 22 types in the fully adjusted models (Supplementary Table 1). For NSA recipients we observed significant association in 9 of 22 health professional types in age and sex adjusted models, reducing to 4 in the fully adjusted models (Supplementary Table 2). The magnitude of the observed associations that remained statistically significant were also attenuated. For example, the prevalence ratio for psychologist in DSP recipients was 4.20 (95%CI: 3.41–5.17) in age and sex adjusted models but attenuated to 1.54 (95%CI: 1.15–2.06) in fully adjusted models. Similar patterns of attenuation were observed for the incidence of HSU and the prevalence of hospital admission, emergency department attendance, medication and supplement use. Details of two fully adjusted models for GP and Dentist prevalence ratios are included in Supplementary Tables 5 and 6, to illustrate the nature of these effects and the impact of including individual confounders. In the GP model, people with poorer self-assessed health, more severe core activity limitation, with private health insurance, over 55 years of age, living in major cities and with more comorbidities had a statistically significantly greater prevalence of consultations with General Practitioners. In the Dentist model, financial resources was an additional significant confounder while severity of disability was not statistically significant.

## Discussion

This study confirms and extends knowledge of the links between working age income support benefit receipt and health service utilisation. Compared to wage earners, Australian working age adults receiving disability or unemployment benefits were more likely to attend consultations with a range of healthcare professionals, have more frequent consultations, and reported higher rates of prescription medicine use. Multiple of these associations were observed to be statistically significant even after accounting for a range of predisposing, enabling and need factors that have been linked with health service use. The largest effects were observed in DSP and NSA groups for psychologists and social workers, suggestive of an increased need for mental health support among Australian income support recipients. This is consistent with a number of prior studies that have reported adverse mental health consequences of disability benefit receipt and transitioning between income support benefits [3, 5]. For multiple other types of health service, including hospital attendance and admission, some health professional consultations, and supplement use, no statistically significant differences were observed between wage earners and those receiving disability or unemployment benefits in adjusted models.

Our findings also suggest that HSU among working age benefit recipients is influenced both by burden of disease and by financial resources. For example, the presence of multiple comorbid conditions, severity of disability and self-assessed health were associated with a higher prevalence of General Practitioner consultations. The prevalence of accessing health services such as GP consultations was also negatively associated with financial resources with those having private health insurance having a higher prevalence of HSU. These findings reflect the well described social and economic gradients in health [19].

Overall, the inclusion of enabling and need factors as confounders in regression models substantially attenuated the relationship between income support group (exposure) and health service use (outcome). For hospital admission and emergency department attendance, inclusion of these factors eliminated the associations observed when only predisposing factors age and sex were included in baseline models. For health professional consultations, the number of significant associations observed were reduced but some remained significant, albeit of lesser magnitude. With respect to medication and supplement use, only the incidence of medication use in DSP recipients remained statistically significantly associated with group status following inclusion of these factors in regression models.

### Implications and future research

We observe significant differences in HSU between working age benefit recipients and wage earners. While much of this difference is explained by health and disability status, and demographic factors such as age, some significant effects remain after adjusting for these factors. Overall, these findings indicate the substantial underlying burden of disease and disability in Australians with work-limiting disability and the unemployed, and suggest that involvement in income support programs may contribute to additional demand for some health services, notably primary care and psychological and social support. This additional demand may be due to a higher rate of psychological and social issues among benefit recipients that requires greater access to these services, or by government programs that reduce barriers to accessing these services for people with significant health conditions. For example, Australian DSP recipients are eligible to receive a government funded ‘health care card’ that provides discounted access to General Practitioners and discounted medicines. The additional HSU is unlikely to be due to impositions of the benefit regime as the Australian income support schemes do not typically mandate ongoing treatment or care, or continued evidence of medical impairment or disability.

Combined, more than 1.45 million Australians received either the DSP or the NSA at the time this analysis was undertaken. That number has increased to over 2 million people during the COVID-19 pandemic, accounting for approximately 17% of the Australian working age population. Australian governments continually tightened access to disability income support benefits with the objective of restricting growth in expenditure and encouraging people to seek paid employment [20]. For instance, requiring most DSP applicants to participate in job seeking or training for a period of 18 months before applying for the DSP. These reforms have reduced access to the DSP and increased the rate of unemployment benefit receipt in working-age Australians with work disabling medical conditions and disability [20]. In other nations such reforms have been linked with adverse health outcomes and significantly impacted determinants of health such as engagement in work. Policies that restricted access to disability benefits in Denmark and Sweden were linked with significantly increased odds of unemployment among people with moderate and severe medical conditions [6] while a program of re-assessing the work capacity of English disability benefit recipients was associated with community mental health impacts including increased suicide, worse mental health and increased prescribing of anti-depressant medicines [7]. Our findings are consistent with these international studies. The descriptive (unadjusted) data in the current study indicates much poorer health and higher HSU for NSA recipients. While this group do not have higher prevalence of GP service use in fully adjusted models, they do report a higher incidence in adjusted models. This suggests that those NSA recipients that do consult with GPs do so more frequently, suggestive of a subset with poor health.

As noted, we observe significant associations between demographic, social and health or disability-related factors and healthcare use. These findings are consistent with the extant literature on the role of socioeconomic factors in healthcare use which observe increased odds of HSU among people with lower incomes, those living in more deprived neighbourhoods and those with less formal education [21]. Our findings are also consistent with studies that observe a relationship between greater disability or functional limitation and increased health care utilisation [22]. We extend these findings by demonstrating a greater prevalence of consultation with some practitioners linked to income support receipt status, after adjusting for these other factors.

There is substantial global evidence of the link between health and the ability to find and maintain work [12, 23]. It follows that effective health service delivery will support improvements in the ability of people receiving income support benefits to participate in job finding and employment. We have noted the potential for greater coordination between health and welfare systems in Australia. There are multiple ways that this coordination may be achieved. For example, it may be feasible to identify the individual health needs of benefit recipients at the point of entry to the welfare system, and then deliver targeted health services that address those needs. Such approaches are being trialled in other Australian benefit systems such as workers’ compensation [24]. It may also be possible to enhance the health system capacity to screen for the social determinants of health, for which a number of screening tools have been developed [25]. These screening approaches may also be extended to social interventions, using models of social prescribing, which have been developed to address the social determinants of health [26]. Our observation of increased use of psychological and social work services amongst benefit recipients demonstrates a need for such services. Future studies should examine the effectiveness of health service delivery to benefit recipients and the impact on health status and ability to engage in employment. Longitudinal studies would provide particularly valuable information, and such studies may be feasible using linked administrative data or a prospective cohort design. It will also be valuable to examine sub-groups of DSP and NSA recipients to identify those with the greatest potential for improvement in health status. Such analyses may provide information to support the targeted delivery of services and supports based on demographic, health or other characteristics.

Since the completion of this study significant changes to both healthcare and government income support systems have taken place in the Australian and international context, in response to the COVID-19 pandemic. In Australia the number of people receiving the unemployment benefit has doubled between March and June 2020 [27], a temporary payment has been introduced to ensure working age people remain attached to their employer during the pandemic, and the government has increased the amount of payment to the unemployed. We have also seen an unprecedented strain on our health care system, disproportionately from those in lower socioeconomic groups [28]. These new developments emphasise the strong interconnection between individual and community health, the operation of income support and healthcare systems.

### Strengths and limitations

Study strengths include the use of a large national sample, data collection using standardised coding schema, and availability of detailed service data across multiple healthcare settings. Availability of data on a range of predisposing, enabling and need factors enabled statistical adjustment of estimates. Limitations include the self-report survey methodology with potential for recall bias, and the potential for social desirability bias to have influenced group inclusion (i.e., stigma associated with receipt of government benefits may discourage some participants from reporting benefit income). There may also be response biases in the sample, as people who are ill and disabled may be less likely to participate in surveys. Due to data limitations we were not able to model the impact of benefit means-testing. Models were adjusted for reported extent of activity limitation and disability status, but not for proxy response, which may be an additional indicator of disability severity. In total, *N* = 12 (2%) of DSP recipients had help completing the survey, as did 2% of NSA recipients and 0.3% of the wage earners group.

## Conclusion

This study provides evidence that better coordination between the Australian public healthcare and income support systems is needed. People receiving unemployment and disability benefits use more health services than wage earners across primary and community health services, and prescription medicines. The study also provides evidence that income support is an independent predictor of health service use, as we observe higher prevalence and incidence of HSU after adjusting for a range of demographic, financial and health/disability factors. Greater coordination between service provision in the health and income support systems may improve health, reduce health service use, and improve work ability in unemployed and working age Australians.

## Supplementary Information

**Additional file 1.**

## Data Availability

The data that support the findings of this study are available from the Australian Bureau of Statistics but restrictions apply to the availability of these data, which were used under license for the current study, and so are not publicly available.

## Abbreviations

CI: Confidence Intervals
DSP: Disability Support Pension
GP: General Practitioner
HSU: Health Service Use
IRR: Incident Rate Ratio
NSA: Newstart Allowance
PR: Prevalence Ratio
