# Supplementary material for: Health service use of Australian unemployment and disability benefit recipients: a national, cross-sectional study
Source: BMC Health Serv Res. 2021 Mar 19;21:249. doi: 10.1186/s12913-021-06255-0 (PMC7977490; doi:10.1186/s12913-021-06255-0)
Supplement: Supplementary file 1 — Additional file 1. [file 12913_2021_6255_MOESM1_ESM.docx]

**SUPPLEMENTARY MATERIAL**

**Reporting of Health Service Use**
Respondents to the NHS were asked whether they had taken any of the following actions in the last 12 months (shown on a prompt card):

- Consulted a GP
- Consulted a specialist
- Consulted a dentist
- Consulted other health professional
- Admitted to hospital as an inpatient
- Visited an outpatient clinic
- Visited emergency/casualty
- Visited a day clinic.

More than one response was allowed. If the respondent indicated they had consulted an 'other health professional' in the last 12 months, they were asked (excluding any time spent in hospital) which other health professional(s) they had consulted, with a list of 24 other health professionals shown no a prompt card:

- Aboriginal Health Worker
- Accredited counsellor
- Acupuncturist
- Alcohol and drug worker
- Audiologist/Audiometrist
- Chemist/Pharmacist (for advice only)
- Chiropodist/Podiatrist
- Chiropractor
- Diabetes educator
- Dietitian/Nutritionist
- Naturopath
- Herbalist
- Hypnotherapist
- Nurse
- Occupational therapist
- Optician/Optometrist/Orthoptist
- Osteopath
- Physiotherapist/Hydrotherapist
- Psychologist
- Radiographer;
- Social worker/Welfare officer
- Sonographer
- Speech therapist/Pathologist
- Other.

More than one response was allowed.

In addition, respondents who had consulted a GP, specialist or dentist, been admitted to hospital as an inpatient, visited an outpatient or day clinic, or visited emergency/casualty in the last 12 months were asked how many times they had done so during that period.

**Reporting of Medication Use**

Respondents were asked for the names or brands of all medications and dietary supplements (vitamins, minerals or natural or herbal supplements) they had taken in the last 2 weeks. Respondents were encouraged to have all of their medications and supplements in front of them during the interview to assist in reporting the information. Interviewers recorded the Australian Register of Therapeutic Goods Administration (TGA) identification number of each medication taken by the respondent. The therapeutic substances people reported were coded as either ‘medications’ or as ‘dietary supplements’. Medications were coded based on their active ingredient(s) and their therapeutic application, to the World Health Organisation Anatomical Therapeutic Chemical (ATC) classification system.

**Supplementary Table 1.** Prevalence Ratios for DSP recipients in comparison to Wage Earners for consultations with Health Professionals in the past 12 months. The table presents results of robust Poisson models adjusted for age and sex only, and robust Poisson models adjusted for all covariates.

|  | **Models Adjusted for Age and Sex only** | | | | **Models Adjusted for all Covariates** | | | |
| --- | --- | --- | --- | --- | --- | --- | --- | --- |
| **Health Professional** | **PR** | **95% CI** | | **p** | **PR** | **95% CI** | | **p** |
| Acupuncturist | 0.77 | 0.40 | 1.49 | 0.44 | 0.78 | 0.35 | 1.73 | 0.54 |
| Audiologist/Audiometrist | 2.34 | 1.27 | 4.31 | 0.01 | 1.02 | 0.48 | 2.14 | 0.97 |
| Chemist (for advice only) | 2.62 | 2.22 | 3.11 | <0.001 | 1.17 | 0.94 | 1.45 | 0.16 |
| Chiropractor | 0.46 | 0.29 | 0.75 | 0.002 | 0.50 | 0.29 | 0.87 | 0.02 |
| Counsellor | 3.93 | 2.70 | 5.72 | <0.001 | 1.17 | 0.72 | 1.90 | 0.53 |
| Dentist | 0.66 | 0.59 | 0.74 | <0.001 | 0.98 | 0.86 | 1.12 | 0.76 |
| Diabetes Educator | 5.08 | 3.45 | 7.47 | <0.001 | 1.23 | 0.70 | 2.19 | 0.47 |
| Dietitian/Nutritionist | 4.31 | 3.19 | 5.83 | <0.001 | 1.49 | 0.97 | 2.27 | 0.07 |
| General Practitioner | 1.10 | 1.08 | 1.12 | <0.001 | 1.04 | 1.01 | 1.07 | 0.02 |
| Naturopath | 0.62 | 0.32 | 1.23 | 0.17 | 0.49 | 0.22 | 1.06 | 0.07 |
| Nurse | 4.11 | 3.16 | 5.34 | <0.001 | 1.26 | 0.89 | 1.77 | 0.19 |
| Occupational Therapist | 3.95 | 2.34 | 6.65 | <0.001 | 0.70 | 0.36 | 1.36 | 0.29 |
| Optician/Optometrist/Orthoptist | 1.51 | 1.20 | 1.91 | 0.001 | 1.19 | 0.87 | 1.62 | 0.28 |
| Osteopath | 0.33 | 0.12 | 0.88 | 0.03 | 0.23 | 0.08 | 0.66 | 0.01 |
| Other Health Professional | 2.39 | 1.87 | 3.05 | <0.001 | 1.27 | 0.92 | 1.74 | 0.14 |
| Physiotherapist/Hydrotherapist | 1.08 | 0.85 | 1.37 | 0.52 | 0.86 | 0.65 | 1.14 | 0.30 |
| Podiatrist | 1.91 | 1.40 | 2.63 | <0.001 | 1.70 | 1.11 | 2.61 | 0.02 |
| Psychologist | 4.20 | 3.41 | 5.17 | <0.001 | 1.54 | 1.15 | 2.06 | 0.004 |
| Radiographer | 1.82 | 1.40 | 2.38 | <0.001 | 0.75 | 0.54 | 1.05 | 0.09 |
| Social Worker/Welfare Officer | 20.01 | 12.99 | 30.84 | <0.001 | 3.58 | 1.79 | 7.16 | <0.001 |
| Sonographer | 1.51 | 0.83 | 2.77 | 0.18 | 0.71 | 0.34 | 1.47 | 0.35 |
| Specialist | 1.65 | 1.53 | 1.78 | <0.001 | 1.19 | 1.07 | 1.31 | 0.001 |

PR – Prevalence ratios, 95% CI – 95% confidence interval

**Supplementary Table 2.** Prevalence Ratios for NSA recipients in comparison to Wage Earners for consultations with Health Professionals in the past 12 months. The table presents results of robust Poisson models adjusted for age and sex only, and Robust Poisson models adjusted for all covariates.

| **Health Professional** | **Models Adjusted for Age and Sex only** | | | | **Models Adjusted for all Covariates** | | | |
| --- | --- | --- | --- | --- | --- | --- | --- | --- |
|  | **PR** | **95% CI** | | **p** | **PR** | **95% CI** | | **p** |
| Acupuncturist | 0.30 | 0.09 | 0.93 | 0.04 | 0.39 | 0.11 | 1.36 | 0.14 |
| Audiologist/Audiometrist | 1.35 | 0.48 | 3.77 | 0.57 | 0.99 | 0.33 | 2.96 | 0.99 |
| Chemist (for advice only) | 1.30 | 0.99 | 1.69 | 0.06 | 0.92 | 0.71 | 1.21 | 0.56 |
| Chiropractor | 0.49 | 0.28 | 0.84 | 0.01 | 0.69 | 0.39 | 1.20 | 0.19 |
| Counsellor | 2.66 | 1.68 | 4.22 | <0.001 | 1.32 | 0.78 | 2.22 | 0.31 |
| Dentist | 0.71 | 0.63 | 0.81 | <0.001 | 1.07 | 0.94 | 1.22 | 0.33 |
| Diabetes Educator | 1.36 | 0.60 | 3.10 | 0.46 | 0.62 | 0.26 | 1.48 | 0.28 |
| Dietitian/Nutritionist | 1.27 | 0.72 | 2.25 | 0.41 | 0.77 | 0.42 | 1.42 | 0.40 |
| General Practitioner | 1.03 | 0.99 | 1.07 | 0.09 | 1.02 | 0.98 | 1.06 | 0.43 |
| Naturopath | 0.73 | 0.36 | 1.47 | 0.38 | 0.75 | 0.36 | 1.58 | 0.45 |
| Nurse | 2.07 | 1.42 | 3.02 | <0.001 | 1.23 | 0.83 | 1.72 | 0.30 |
| Occupational Therapist | 1.32 | 0.53 | 3.28 | 0.54 | 0.51 | 0.20 | 1.32 | 0.16 |
| Optician/Optometrist/Orthoptist | 0.51 | 0.31 | 0.82 | 0.01 | 0.58 | 0.35 | 0.95 | 0.03 |
| Osteopath | 0.37 | 0.12 | 1.17 | 0.09 | 0.41 | 0.13 | 1.34 | 0.14 |
| Other Health Professional | 1.26 | 0.87 | 1.82 | 0.22 | 1.02 | 0.68 | 1.51 | 0.94 |
| Physiotherapist/Hydrotherapist | 0.52 | 0.35 | 0.78 | 0.002 | 0.66 | 0.44 | 0.99 | 0.04 |
| Podiatrist | 0.66 | 0.35 | 1.28 | 0.22 | 0.84 | 0.43 | 1.63 | 0.61 |
| Psychologist | 2.50 | 1.91 | 3.29 | <0.001 | 1.42 | 1.04 | 1.95 | 0.03 |
| Radiographer | 1.24 | 0.84 | 1.82 | 0.28 | 0.98 | 0.65 | 1.50 | 0.94 |
| Social Worker/Welfare Officer | 8.45 | 4.93 | 14.50 | <0.001 | 3.09 | 1.61 | 5.93 | 0.001 |
| Sonographer | 0.85 | 0.35 | 2.06 | 0.72 | 0.70 | 0.27 | 1.81 | 0.47 |
| Specialist | 1.13 | 0.99 | 1.28 | 0.06 | 1.09 | 0.96 | 1.25 | 0.18 |

PR – Prevalence ratios, 95% CI – 95% confidence interval

**Supplementary Table 3.** Incidence Rate Ratios for DSP and NSA recipients in comparison to Wage Earners for frequency of consultations with various Health Practitioners and Hospital Attendance/Admission in the past 12 months, and for number of different medications or supplements taken in the past 2 weeks. The table presents results of negative binomial models adjusted for age and sex only, and negative binomial models adjusted for all covariates.

| **Health Action** | **Group** | **Models Adjusted for Age and Sex only** | | | | **Models Adjusted for all Covariates** | | | |
| --- | --- | --- | --- | --- | --- | --- | --- | --- | --- |
|  |  | **IRR** | **95% CI** | | **p** | **IRR** | **95% CI** | | **p** |
| General Practitioner | DSP | 2.37 | 2.21 | 2.54 | <0.001 | 1.27 | 1.17 | 1.37 | <0.001 |
| Dentist |  | 0.98 | 0.86 | 1.11 | 0.75 | 1.04 | 0.88 | 1.22 | 0.64 |
| Specialist |  | 2.82 | 2.37 | 3.37 | <0.001 | 1.49 | 1.21 | 1.84 | <0.001 |
| Admitted to Hospital |  | 3.74 | 2.97 | 4.71 | <0.001 | 1.07 | 0.79 | 1.45 | 0.66 |
| Visited Emergency |  | 4.51 | 3.55 | 5.74 | <0.001 | 1.02 | 0.76 | 1.39 | 0.88 |
| Medications |  | 3.78 | 3.07 | 3.72 | <0.001 | 1.49 | 1.34 | 1.66 | <0.001 |
| Supplements |  | 0.81 | 0.71 | 0.93 | 0.002 | 0.92 | 0.78 | 1.09 | 0.36 |
| General Practitioner | NSA | 1.56 | 1.43 | 1.69 | <0.001 | 1.11 | 1.02 | 1.20 | 0.02 |
| Dentist |  | 0.79 | 0.68 | 0.93 | 0.003 | 1.03 | 0.87 | 1.22 | 0.72 |
| Specialist |  | 1.30 | 1.05 | 1.60 | 0.02 | 1.09 | 0.87 | 1.36 | 0.45 |
| Admitted to Hospital |  | 1.96 | 1.47 | 2.62 | <0.001 | 1.21 | 0.89 | 1.66 | 0.22 |
| Visited Emergency |  | 1.76 | 1.30 | 2.39 | <0.001 | 0.94 | 0.68 | 1.30 | 0.72 |
| Medications |  | 1.61 | 1.14 | 1.82 | <0.001 | 1.12 | 0.99 | 1.27 | 0.07 |
| Supplements |  | 0.77 | 0.66 | 0.91 | 0.002 | 0.93 | 0.79 | 1.11 | 0.44 |

IRR – Incidence rate ratios, 95% CI – 95% confidence interval

**Supplementary Table 4.** Prevalence Ratios for DSP and NSA recipients in comparison to Wage Earners for being admitted to hospital or visiting emergency in the past 12 months, and for taking a medication or supplement in the past 2 weeks. The table presents results of robust Poisson models adjusted for age and sex only and robust Poisson models adjusted for all covariates.

| **Health Action** | **Group** | **Models Adjusted for Age and Sex only** | | | | **Models Adjusted for all Covariates** | | | |
| --- | --- | --- | --- | --- | --- | --- | --- | --- | --- |
|  |  | **PR** | **95% CI** | | **p** | **PR** | **95% CI** | | **p** |
| Admitted to Hospital | DSP | 2.46 | 2.08 | 2.89 | <0.001 | 1.17 | 0.94 | 1.47 | 0.16 |
| Visited Emergency |  | 2.35 | 1.98 | 2.79 | <0.001 | 0.88 | 0.70 | 1.11 | 0.29 |
| Medications |  | 1.64 | 1.57 | 1.71 | <0.001 | 1.15 | 1.08 | 1.23 | <0.001 |
| Supplements |  | 0.88 | 0.79 | 0.97 | 0.01 | 1.00 | 0.88 | 1.13 | 0.97 |
| Admitted to Hospital | NSA | 1.63 | 1.30 | 2.06 | <0.001 | 1.20 | 0.92 | 1.55 | 0.18 |
| Visited Emergency |  | 1.61 | 1.28 | 2.02 | <0.001 | 0.97 | 0.75 | 1.25 | 0.81 |
| Medications |  | 1.17 | 1.07 | 1.27 | <0.001 | 0.99 | 0.90 | 1.08 | 0.75 |
| Supplements |  | 0.80 | 0.70 | 0.91 | 0.001 | 0.94 | 0.82 | 1.08 | 0.41 |

PR – Prevalence ratios, 95% CI – 95% confidence interval

**Supplementary Table 5.** Complete results for the Robust Poisson model for the prevalence of consulting with a General Practitioner in the past 12 months

| **Variable** | **PR** | **95% CI** | | **p** |
| --- | --- | --- | --- | --- |
| **Group** |  |  |  |  |
| Wage Earners | 1 (reference) |  |  |  |
| Disability Support Pension | 1.04 | 1.01 | 1.07 | 0.02 |
| Newstart Allowance | 1.02 | 0.98 | 1.06 | 0.43 |
| **Age in Years** |  |  |  |  |
| 18-24 | 1.01 | 0.97 | 1.05 | 0.54 |
| 25-34 | 1.02 | 0.99 | 1.05 | 0.20 |
| 35-44 | 1 (reference) |  |  |  |
| 45-54 | 1.01 | 0.98 | 1.03 | 0.67 |
| 55-64 | 1.05 | 1.03 | 1.08 | <0.001 |
| **Sex** |  |  |  |  |
| Male | 1 (reference) |  |  |  |
| Female | 1.11 | 1.09 | 1.13 | <0.001 |
| **Highest Level of Education** |  |  |  |  |
| Less than Year 12 | 0.96 | 0.94 | 0.99 | 0.004 |
| Year 12 | 0.98 | 0.96 | 1.01 | 0.28 |
| Certificate or Diploma | 1 (reference) |  |  |  |
| Bachelor Degree | 1.00 | 0.97 | 1.02 | 0.69 |
| Postgraduate Studies | 0.98 | 0.95 | 1.00 | 0.10 |
| **Private Health Insurance Status** |  |  |  |  |
| Without Private Health Insurance | 0.94 | 0.92 | 0.96 | <0.001 |
| With Private Health Insurance | 1 (reference) |  |  |  |
| **Whether Household could Raise $2000 in an Emergency** |  |  |  |  |
| No | 1.00 | 0.98 | 1.03 | 0.73 |
| Yes | 1 (reference) |  |  |  |
| **Remoteness** |  |  |  |  |
| Major cities of Australia | 1 (reference) |  |  |  |
| Inner regional Australia | 0.96 | 0.94 | 0.99 | 0.002 |
| Other | 0.97 | 0.95 | 0.99 | 0.02 |
| **Self-Assessed Health** |  |  |  |  |
| Excellent | 0.94 | 0.92 | 0.97 | <0.001 |
| Very good | 1 (reference) |  |  |  |
| Good | 1.01 | 0.99 | 1.04 | 0.16 |
| Fair | 1.05 | 1.02 | 1.07 | 0.001 |
| Poor | 1.05 | 1.02 | 1.09 | 0.004 |
| **Disability Status** |  |  |  |  |
| No disability or long-term health condition | 1 (reference) |  |  |  |
| No limitation or specific restriction | 1.05 | 1.03 | 1.08 | <0.001 |
| Schooling/employment restriction only | 1.06 | 1.03 | 1.09 | <0.001 |
| Mild/moderate core activity limitation | 1.03 | 1.01 | 1.06 | 0.01 |
| Severe/profound core activity limitation | 1.04 | 1.01 | 1.08 | 0.01 |
| **Number of ICD-10 Conditions** |  |  |  |  |
| 0-4 | 1 (reference) |  |  |  |
| 5-9 | 1.12 | 1.10 | 1.14 | <0.001 |
| 10-14 | 1.12 | 1.10 | 1.15 | <0.001 |
| 15+ | 1.10 | 1.06 | 1.13 | <0.001 |

PR – Prevalence ratios, 95% CI – 95% confidence interval, DSP – Disability Support Pension, NSA – Newstart Allowance

**Supplementary Table 6.** Complete results for the robust Poisson model for the prevalence of consulting with a dentist in the past 12 months.

| **Variable** | **PR** | **95% CI** | | **p** |
| --- | --- | --- | --- | --- |
| **Group** |  |  |  |  |
| Wage Earners | 1 (reference) |  |  |  |
| Disability Support Pension | 0.98 | 0.86 | 1.12 | 0.76 |
| Newstart Allowance | 1.07 | 0.94 | 1.22 | 0.33 |
| **Age in Years** |  |  |  |  |
| 18-24 | 1.00 | 0.91 | 1.10 | 0.95 |
| 25-34 | 0.92 | 0.86 | 0.98 | 0.01 |
| 35-44 | 1 (reference) |  |  |  |
| 45-54 | 1.03 | 0.97 | 1.10 | 0.28 |
| 55-64 | 1.14 | 1.08 | 1.21 | <0.001 |
| **Sex** |  |  |  |  |
| Male | 1 (reference) |  |  |  |
| Female | 1.16 | 1.11 | 1.21 | <0.001 |
| **Highest Level of Education** |  |  |  |  |
| Less than Year 12 | 0.88 | 0.82 | 0.95 | 0.001 |
| Year 12 | 1.01 | 0.94 | 1.08 | 0.82 |
| Certificate or Diploma | 1 (reference) |  |  |  |
| Bachelor Degree | 1.10 | 1.04 | 1.16 | 0.001 |
| Postgraduate Studies | 1.11 | 1.05 | 1.18 | 0.001 |
| **Private Health Insurance Status** |  |  |  |  |
| Without Private Health Insurance | 0.59 | 0.56 | 0.63 | <0.001 |
| With Private Health Insurance | 1 (reference) |  |  |  |
| **Whether Household could Raise $2000 in an Emergency** |  |  |  |  |
| No | 0.79 | 0.72 | 0.87 | <0.001 |
| Yes | 1 (reference) |  |  |  |
| **Remoteness** |  |  |  |  |
| Major cities of Australia | 1 (reference) |  |  |  |
| Inner regional Australia | 0.96 | 0.91 | 1.02 | 0.22 |
| Other | 0.89 | 0.83 | 0.95 | <0.001 |
| **Self-Assessed Health** |  |  |  |  |
| Excellent | 1.07 | 1.01 | 1.12 | 0.01 |
| Very good | 1 (reference) |  |  |  |
| Good | 0.91 | 0.86 | 0.96 | 0.001 |
| Fair | 0.99 | 0.91 | 1.07 | 0.76 |
| Poor | 0.88 | 0.75 | 1.04 | 0.13 |
| **Disability Status** |  |  |  |  |
| No disability or long-term health condition | 1 (reference) |  |  |  |
| No limitation or specific restriction | 1.04 | 0.98 | 1.10 | 0.20 |
| Schooling/employment restriction only | 1.05 | 0.94 | 1.16 | 0.39 |
| Mild/moderate core activity limitation | 0.99 | 0.92 | 1.07 | 0.82 |
| Severe/profound core activity limitation | 1.08 | 0.94 | 1.25 | 0.27 |
| **Number of ICD-10 Conditions** |  |  |  |  |
| 0-4 | 1 (reference) |  |  |  |
| 5-9 | 1.15 | 1.10 | 1.21 | <0.001 |
| 10-14 | 1.18 | 1.07 | 1.30 | 0.001 |
| 15+ | 1.48 | 1.25 | 1.76 | <0.001 |

PR – Prevalence ratios, 95% CI – 95% confidence interval, DSP – Disability Support Pension, NSA – Newstart Allowance
